# Comprehensive Genome-Wide Characterization of L-Type Lectin Receptor-like Kinase (L-LecRLK) Genes in Wheat (*Triticum aestivum* L.) and Their Response to Abiotic Stress

**DOI:** 10.3390/plants14121884

**Published:** 2025-06-19

**Authors:** Wan Zhao, Fuyan Zhang, Jiahuan Wang, Shuai Fang, Zhongjie Cheng, Xuhui Ma, Jialin Fan, Zhaoshi Xu, Xiaojie Chen

**Affiliations:** 1 Institute of Chemistry, Henan Academy of Sciences, Zhengzhou 450002, China; 2 Isotope Institute Co., Ltd., Henan Academy of Sciences/Henan Key Laboratory of Nuclear Agricultural Sciences, Zhengzhou 450015, China; 3 State Key Laboratory of Crop Gene Resources and Breeding, Institute of Crop Sciences, Chinese Academy of Agricultural Sciences (CAAS), Beijing 100081, China

**Keywords:** *Triticum aestivum* L., L-type lectin receptor-like kinase, phylogeny, gene expression, abiotic stress

## Abstract

L-type lectin receptor-like kinases (L-LecRLKs) play key roles in plant responses to environmental stresses and the regulation of growth and development. However, comprehensive studies of the L-LecRLK gene family in wheat (*Triticum aestivum* L.) are still limited. In this study, 248 L-LecRLK candidate genes were identified in wheat, which is the largest number reported in any species to date. Phylogenetic analysis grouped these genes into four clades (I–IV), with Group IV exhibiting significant monocot-specific expansion. Gene duplication analysis revealed that both whole-genome/segmental and tandem duplications contributed to family expansion, while Ka/Ks ratio analysis suggested that the genes have undergone strong purifying selection. The *TaL-LecRLK* genes displayed diverse exon-intron structures and conserved motif compositions. Promoter analysis revealed a *cis*-element associated with hormone signaling and abiotic stress responses. Transcriptome profiling showed that *TaL-LecRLKs* exhibit tissue- and stage-specific expression patterns. RNA-Seq data revealed that, under drought and heat stress conditions, *TaL-LecRLK35-3D* and *TaL-LecRLK67-6B* exhibited synergistic expression patterns, whereas *TaL-LecRLK67-6A* demonstrated antagonistic expression. A qRT-PCR further demonstrated that six *TaL-LecRLKs* may function through ABA-independent regulatory mechanisms. These findings provide valuable gene candidates for stress-resistant wheat breeding and shed light on the evolution and functional diversity of L-LecRLKs in plants.

## 1. Introduction

Wheat (*Triticum aestivum* L.) is the most widely cultivated cereal crop globally, serving not only as a primary food source for humans but also as a crucial feedstock for livestock and an important raw material for various industries, occupying a central position in food security and the global economy [1,2]. Common wheat has undergone two rounds of polyploidization during its evolution, resulting in an allohexaploid genome of approximately 15 Gb [3,4]. This large and complex genome presents significant challenges for genomic research and breeding efforts. The release of a high-quality reference genome for the hexaploid cultivar ‘Chinese Spring’ by the International Wheat Genome Sequencing Consortium (IWGSC) in 2018 [5] laid the groundwork for advanced molecular breeding. Subsequent multi-omics analyses based on this reference revealed dynamic expression of homologous genes across developmental stages and stress conditions [6], facilitating gene cloning and functional characterization for key agronomic traits.

Lectin receptor-like kinases (LecRLKs) form a distinct subgroup of receptor-like protein kinases (RLKs) unique to higher plants, characterized by extracellular lectin motifs’ domains [7,8]. A typical LecRLK protein comprises three domains: an extracellular lectin domain for ligand recognition, a transmembrane region for membrane anchoring, and an intracellular kinase domain responsible for signal transduction [9]. Based on extracellular lectin domain structures and phylogenetic relationships, LecRLKs are categorized into three subtypes: C-type, G-type, and L-type [7,10]. C-type LecRLKs are primarily found in mammals and contain a calcium-dependent carbohydrate-binding lectin domain [11]. Currently, only a single member has been identified in various plant species, such as *Arabidopsis*, rice, barley, foxtail millet, and soybean [12,13,14,15]. G-type LecRLKs, which were previously known as S-domain RLKs or B-type LecRLKs, have a lectin domain characterized by a *β*-barrel structure and are predicted to bind α-D-mannose specificity [8,16]. Many G-type LecRLKs also contain an Epidermal Growth Factor (EGF) and/or PAN motifs, both of which are absent in L-type and C-type LecRLKs [17]. The EGF motif is rich in cysteine residues and is potentially involved in the formation of disulfide bonds, while the PAN motif mediates protein–protein and protein–carbohydrate interactions [16,18]. L-type LecRLKs (L-LecRLKs), the extracellular domains, resemble legume lectins and adopt a *β*-sandwich fold structure [19]. These domains contain hydrophobic cavities that bind various hydrophobic ligands such as polysaccharides, phytohormones, and PAMPs [7,20], and their carbohydrate-binding activity is stabilized by Ca^2+^/Mn^2+^ coordination [10]. The transmembrane regions (TMRs) of LecRLKs typically consist of 18–25 amino acids and exhibit low conservatism [19]. Notably, not all LecRLKs contain a TMR, nor do all members possess only one single transmembrane region [21,22]. Although the TMR is non-essential for the structural integrity of L-LecRLK proteins and shows low conservatism, it is crucial for their kinase activity. Studies demonstrated that a single amino acid mutation in the TMR can lead to a loss of kinase function [23]. The kinase domains of LecRLKs are highly conserved, typically comprising 200–300 amino acids and containing phosphorylation binding sites. These domains are located in the cytoplasm and are primarily responsible for transmitting external signals [24]. The N-terminus of the kinase domain contains a short GxGxxG (x represents any amino acid) amino acid sequence motif, which can influence nucleotide binding, while the C-terminus, consisting of 43 to 66 amino acids, is essential for the kinase’s catalytic activity [24].

The sequence and structural diversity of LecRLKs underlie the molecular basis for their functional diversity. Research demonstrated that LecRLKs function as important signaling receptors that recognize extracellular carbohydrate ligands and play central roles in plant immunity and development [7]. In immune defense, LecRLKs function as pattern recognition receptors (PRRs) that detect pathogen-associated molecular patterns (PAMPs). For example, following infection with *Ralstonia solanacearum*, the *Arabidopsis* lectin receptor-like kinase LORE is activated by phosphorylation at residue S761, initiating a phosphorelay that activates reactive oxygen species’ (ROS) production and cell wall lignification, thereby contributing to basal resistance in the xylem [25]. In the developmental process, OsDAF1 in rice interacts with OsINP1 to regulate pollen aperture formation [26], while Arabidopsis LecRK-VIII.2, acting as an upstream component of the MAPK signaling pathway, modulates silique number, seed size, and seed number to determine seed yield, demonstrating considerable promise for crop improvement [27]. Recent studies increasingly indicate that LecRLKs, particularly L-LecRLKs, play significant roles in various abiotic stress responses. For example, overexpression of the rice *OsLec-RLK* gene in pigeon pea significantly enhanced plant salt stress tolerance [28]. The transgenic plants exhibited superior physiological and biochemical traits (such as higher K^+^/Na^+^ ratio, enhanced antioxidant enzyme activity) and yield performance under salt stress [28]. Furthermore, studies revealed that PaLectinL7 can enhance salt tolerance in sweet cherry via interaction with the lignin-metabolizing enzyme PaCAD1 to regulate lignin deposition [29]. Moreover, expression profiles and qRT-PCR experiments across multiple species further confirmed the involvement of L-LecRLKs in responses to drought, salt, and temperature stress [30,31,32,33]. Despite wheat’s global importance, few studies have systematically examined the L-LecRLK gene family, and many annotations remain incomplete or imprecise [34].

Given the increasing impact of climate change and the plateauing of genetic gains in wheat, the identification of novel stress-responsive genes is essential for advancing molecular breeding. L-LecRLKs, as central regulators of stress adaptation and development, offer promising targets. In this study, we systematically analyzed the wheat *L-LecRLK* gene family, integrating phylogenetic reconstruction, evolutionary analysis, and spatiotemporal expression profiling. Our aim was to refine the classification framework of wheat L-LecRLKs, uncover evolutionary patterns, and link gene expression dynamics with potential biological functions. These findings will provide a theoretical foundation for future functional genomics research and facilitate the development of stress-resilient wheat cultivars through molecular breeding.

## 2. Results

### 2.1. Identification and Characterization of TaL-LecRLK Genes in Wheat

A total of 248 candidate genes encoding L-type lectin receptor-like kinases (L-LecRLKs) were identified in the wheat genome. These genes were designated *TaL-LecRLK1* to *TaL-LecRLK88* based on chromosomal location and homology (Tables S1 and S2). In-paralogous genes originating from the same genome were differentiated through sequential numbering. For instance, *TaL-LecRLK72-6A1*, *TaL-LecRLK72-6A2*, and *TaL-LecRLK72-6A3* were separately identified. Although all 248 candidate genes encoded the same class of protein kinase, their physicochemical properties varied widely. The encoded proteins ranged from 274 (TaL-LecRLK12-2B) to 1054 (TaL-LecRLK24-2A) amino acids in length, with an average of 663 amino acids. Corresponding molecular weights spanned from 31.2 (TaL-LecRLK12-2B) to 115.7 (TaL-LecRLK24-2A) kDa, and theoretical isoelectric points (*p*I) ranged from 5.27 (TaL-LecRLK46-4D) to 10.22 (TaL-LecRLK78-7D) (Table S1). Approximately 67.7% (168/248) of these proteins were classified as acidic. Instability indices ranged from 25.35 (TaL-LecRLK35-3A2) to 61.68 (TaL-LecRLK78-7D), with 74.2% (148/248) categorized as stable and 25.8% (64/248) as unstable at the sequence level. The aliphatic index spanned from 68.0 (TaL-LecRLK78-7D) to 104.16 (TaL-LecRLK12-2B), while the Grand Average of Hydropathicity (GRAVY) values ranged from −0.379 (TaL-LecRLK39-3B) to 0.092 (TaL-LecRLK5-1A) (Table S1).

Subcellular localization predictions indicated that 85 proteins localized to the cell membrane, while 43 were distributed across the cytoplasm, cell membrane, and various organelles. Thirty-five proteins were present in both the cytoplasm and cell membrane, another 35 were localized to the cytoplasm and organelles, and 30 were associated with the cell membrane and membrane-bound organelles such as the endoplasmic reticulum, mitochondria, lysosomes, and vacuoles. Additionally, 18 proteins localized exclusively to the cytoplasm, one to the endoplasmic reticulum and one to both the endoplasmic reticulum and lysosomes/vacuoles (Table S1). Among the proteins, 67.3% (167/248) were predicted to be soluble. Furthermore, 35 proteins contained mitochondrial targeting peptide (MTP) signals, and another 35 were identified as lipid-anchored proteins (Table S1). These findings suggest the involvement of TaL-LecRLK proteins in diverse physiological processes such as signal transduction and cell metabolism.

### 2.2. Phylogenetic Relationships and Classification of TaL-LecRLK Proteins

**Figure 1:**
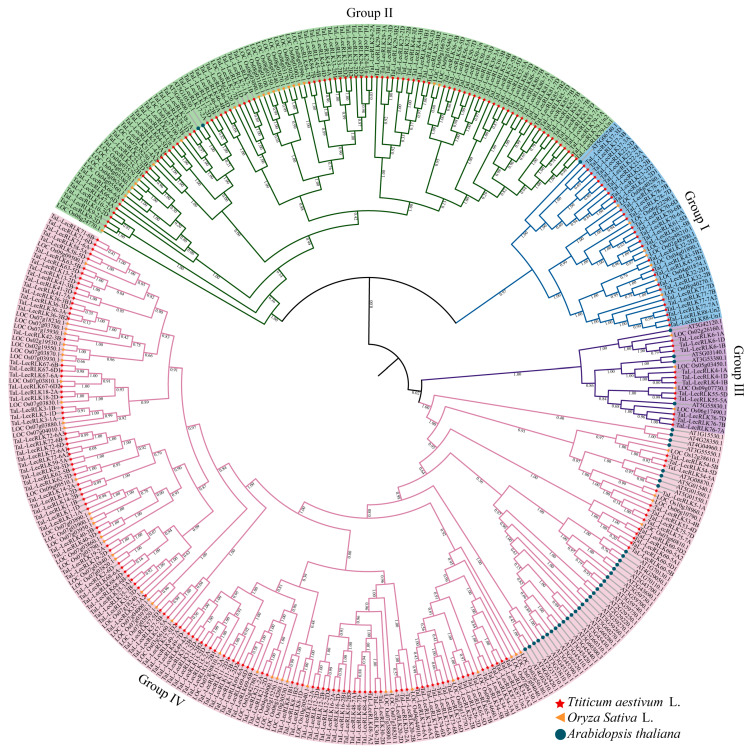
Phylogenetic analysis of L-LecRLK proteins in wheat (*Triticum aestivum* L.), rice (*Oryza sativa* L.), and *Arabidopsis thaliana*. Full-length amino acid sequences were aligned using MUSCLE, and the phylogenetic tree was constructed using the maximum likelihood method in FastTree. Clades are shown in different colors: Group I (green), Group II (blue), Group III (purple), and Group IV (pink). Species are marked by colored shapes: wheat (red star), rice (yellow triangle), and Arabidopsis (green circle).

To investigate the evolutionary relationships of L-LecRLKs across different species, a phylogenetic tree was constructed using 367 homologous sequences, including 248 from wheat (*Triticum aestivum*), 76 from rice (*Oryza sativa*), and 43 from *Arabidopsis thaliana* (Tables S1 and S3; Figure 1). An additional tree was generated exclusively for the 248 wheat TaL-LecRLK proteins (Figure S1).

Based on sequence homology and following the classification used in Arabidopsis and rice [31], the wheat proteins were categorized into four distinct groups: Group I (*n* = 29), Group II (*n* = 83), Group III (*n* = 11), and Group IV (*n* = 125). Groups II and IV were the largest subfamilies, accounting for 84.3% of all *TaL-LecRLK* genes (Figure 1 and Figure S1). The phylogenetic analysis revealed significant expansion in Groups I, II, and IV in wheat compared to Arabidopsis and rice. Several subclades were observed to be species-specific, forming distinct monocot or dicot lineages. This pattern suggests that functional diversification of LecRLK genes may have occurred during evolution to meet species-specific physiological needs.

### 2.3. Gene Structure and Conserved Motif Analysis of TaL-LecRLKs

**Figure 2:**
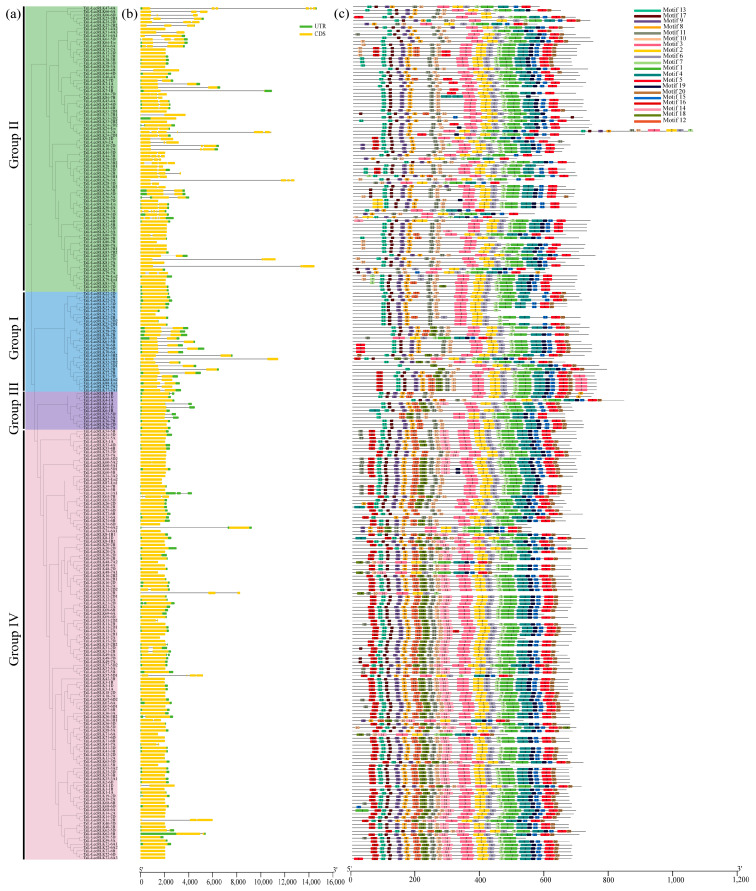
Phylogenetic analysis, conserved motifs, and gene structure of wheat L-LecRLKs. (**a**) Phylogenetic tree of 248 L-LecRLK proteins in wheat constructed based on sequence alignment results; (**b**) gene structures: exons (yellow rectangles), UTRs (blue rectangles), and introns (lines connecting exons). Box and line lengths are proportional to gene length. Intron distribution is summarized in Table 1. (**c**) Motif compositions of 20 conserved motifs identified using MEME. Each motif is represented by a distinct colored box. Detailed motif data are in Table 2.

To explore structural diversity and potential functional divergence, exon-intron structures and conserved motifs were analyzed for all 248 *TaL-LecRLK* genes (Figure 2). Intron counts ranged from 0 to 6 (Table 1), with a significant inverse relationship between intron number and gene abundance. Intronless genes were the most common (148/248; 59.68%), followed by those with one intron (28.2%; 70/248). Only 12.1% of the genes had three or more introns. Within Group IV, 84% (104/125) of the genes were intronless, indicating high evolutionary conservation. In contrast, Group II displayed the greatest intron variability (0–6), suggesting extensive intron gain or loss events (Figure 2a,b; Table 1). Only one gene, *TaL-LecRLK39-3B*, contained six introns, potentially reflecting domain or exon duplication during evolution (Figure 2b; Table 1).

A total of 20 conserved motifs were identified among the TaL-LecRLK proteins, with individual proteins containing between 8 and 24 motifs (Table 2 and Table S1). As illustrated in Figure 2c, proteins sharing similar motif compositions clustered together, indicating potential functional similarities. Highly conserved motifs included motifs 10, 11, 3, 2, 6, 13, 7, 9, and 8 (Figure 2c). Motifs 14 and 16 were specific to Group IV and rarely appeared in other groups, suggesting evolutionary adaptation (Figure 2a,c). Motif 18 was almost entirely absent in Group II but was frequently found in Groups I, III, and IV (Figure 2a,c), further supporting functional divergence among groups.

### 2.4. Chromosomal Localization and Homoeolog Identification of the TaL-LecRLKs

**Figure 3:**
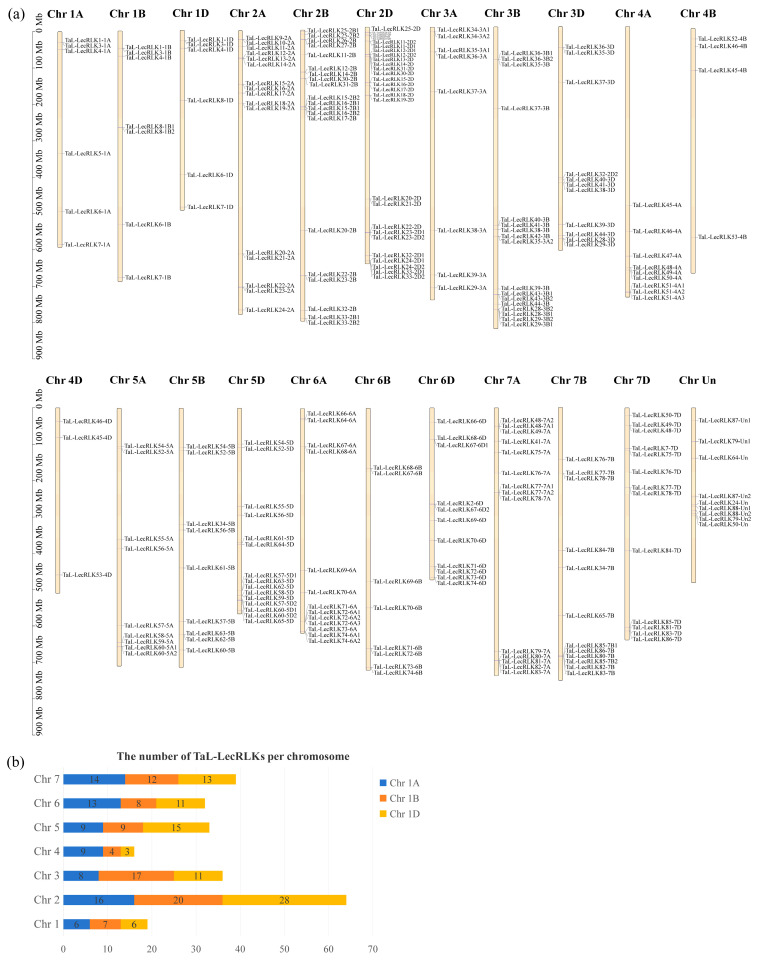
Chromosomal distribution of *TaL-LecRLK* genes. (**a**) Distribution map of 248 genes on wheat chromosomes. Gene names are listed on the right side, scale is in megabases (Mb); (**b**) numbers of *TaL-LecRLK* genes per chromosome (Chr1–Chr7).

The chromosomal positions of the *Ta-LecRLK* genes are listed in Table S1. As illustrated in Figure 3, a heterogeneous distribution pattern was observed for the 248 *TaL-LecRLK* genes across the 21 chromosomes. Chromosome 2D possessed the highest number of *TaL-LecRLK* genes (28), followed by chromosomes 2B (20), 3B (17), and 2A (16). In contrast, chromosomes 4D and 4B contained the fewest, with only 3 and 4 *TaL-LecRLK* genes, respectively. Chromosome group 2 (comprising 2A, 2B, and 2D) possessed the largest total number of *TaL-LecRLK* genes (64), while chromosome group 4 (4A, 4B, and 4D) exhibited the smallest total (16) (Figure 3a,b). The distribution of *TaL-LecRLK* genes among other chromosome groups varied, demonstrating differences among the wheat subgenomes.

To further understand the evolutionary history of the *TaL-LecRLK* genes, homoeologous group analysis was performed (Table 3 and Table S2). It was found that 29% (72/248) of *TaL-LecRLK* genes were organized into complete triads, maintaining strict 1:1:1 orthologous relationships across the A, B, and D homoeologous chromosome groups. This proportion was lower than the 35.8% whole-genome triad frequency [5]. Additionally, higher proportions of homoeolog-specific duplications (n:1:1/1:n:1/1:1:n; 21.8% vs. 5.7%) and single homoeolog losses (1:1:0/1:0:1/0:1:1; 24.6% vs. 13.2%) were identified, as compared to the whole-genome averages (Table 3). However, the frequency of orphan or singleton genes within the *TaL-LecRLK* family (1.6%) was significantly lower than that of the entire wheat genome (37.1%; Table 3).

### 2.5. Duplication and Syntenic Analyses of the L-Type LecRLK Genes

**Figure 4:**
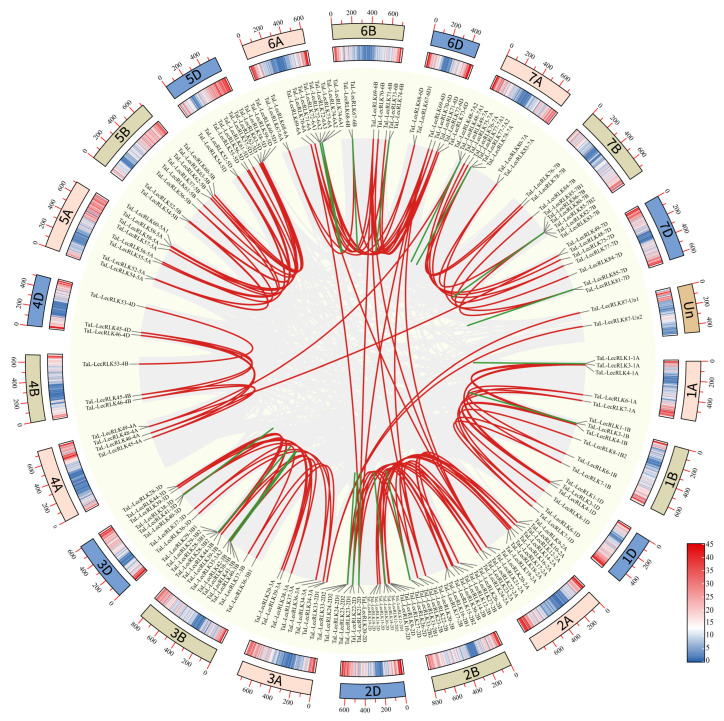
Duplication events of *TaL-LecRLK* genes in wheat. Chromosome numbers are labeled around the circle, with a scale bar in Mb. Segmental duplications are shown as red lines, while tandem duplications are shown as green lines. Gray areas represent syntenic blocks.

To explore the mechanisms driving the expansion of *TaL-LecRLK* genes, synteny analysis was conducted within the wheat genome. A total of 183 genes were located within syntenic blocks, forming 156 pairs of duplicated genes (Figure 4; Table S4). Of the 248 *TaL-LecRLK* genes, approximately 19% (47 genes) were identified as tandem duplicates, these included 5 sets of three tandemly duplicated genes and 22 sets of two tandemly duplicated genes. Additionally, 60% (156/248) of the genes were associated with whole-genome duplication (WGD) or segmental duplication events (Table S4). Notably, chromosome group 2 (2A, 2B, and 2D) exhibited the highest frequency of WGD/segmental duplications. These findings suggest that the polyploidization history of wheat, particularly its hexaploid genome formation, likely contributed to the extensive expansion of the L-type LecRLK gene family through WGD/segmental duplication.

**Figure 5:**
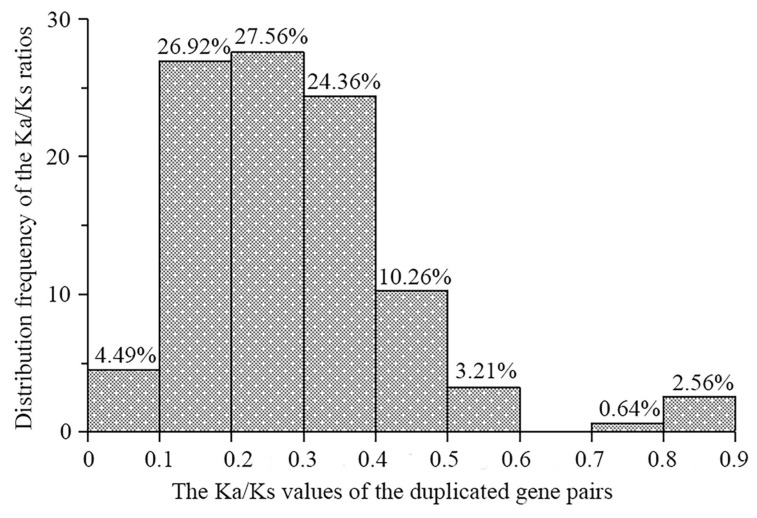
Histogram of pairwise *Ka*/*Ks* ratios for duplicated *TaL-LecRLK* gene pairs.

To assess the evolutionary pressures acting on duplicated *TaL-LecRLK* genes, the ratio of nonsynonymous to synonymous substitutions (Ka/Ks) was calculated for each gene pair. The Ka/Ks ratios ranged from 0.07 to 0.90 (all ratios < 1), with an average of 0.29 (Table S4). As shown in Figure 5, more than 78% of the duplicated gene pairs exhibited Ka/Ks ratios within the 0.1 to 0.3 range. This distribution suggests that these genes underwent strong purifying selection, which acted to conserve their functional stability throughout evolutionary history.

To clarify the evolutionary trajectory of *L-LecRLK* genes across plant species, a genome collinearity analysis was conducted among wheat (*Triticum aestivum* L.), rice (*Oryza sativa*), and *Arabidopsis thaliana*. As shown in Figure S2, only two L-LecRLK loci in *Arabidopsis* exhibited collinearity with two non-TaL-LecRLK loci in the wheat genome, indicating lineage-specific functional divergence of L-LecRLK genes following monocot–dicot divergence. In contrast, a stronger syntenic relationship was observed between the monocot species (rice-wheat), with 16 rice *L-LecRLK* genes corresponding to 45 wheat homologs. In most cases, one rice gene was collinear with 3–5 wheat genes (Figure S2). These results highlight the impact of polyploidization in expanding the *TaL-LecRLK* gene family and help explain the substantially higher number of *L-LecRLK* genes in hexaploid wheat relative to diploid plant species.

### 2.6. Analysis of Cis-Acting Elements in the Promoters of the TaL-LecRLK Genes

To further investigate the potential regulatory functions of *TaL-LecRLK* genes and their involvement in signal transduction pathways, a comprehensive analysis of cis-acting elements in their promoter regions was conducted. A total of 5804 *cis*-acting elements were identified (Table S5). As illustrated in Figure S3, these elements exhibited an uneven distribution pattern across the promoters of the 248 *TaL-LecRLK* genes.

Based on their biological roles, these elements were categorized into three major categories: hormone-responsive elements, environmental adaptation elements, and growth- and development-related elements (Table 4). Hormone-responsive elements represented the largest proportion, accounting for 49.1% of the total. These included elements responsive to five phytohormones: MeJA (methyl jasmonate), ABA (abscisic acid), GA (gibberellin), IAA (auxin), and SA (salicylic acid). Among these, ABREs (ABA-responsive elements) were the most prevalent (28.40%), followed by MeJA-responsive elements—CGTCA-motif (25.9%) and TGACG-motif (25.8%) (Table 4) —suggesting that *TaL-LecRLK* genes may play pivotal roles in ABA and MeJA signaling pathways. Environmental adaptation elements accounted for approximately 41% of the total. These included light-responsive elements (e.g., G-Box, Sp1, GT1-motif), anaerobic induction elements (ARE), drought-inducibility elements (MBS), and low-temperature responsive elements (LTR), indicating that *TaL-LecRLK* genes are potentially involved in diverse environmental stress responses (Table 4). Notably, some *cis*-acting elements exhibited gene-specific distributions. For instance, the light-responsive 4cl-CMA2b element was found exclusively in the *TaL-LecRLK68-6A* promoter, while DRE elements (associated with dehydration, low-temperature, and salt stresses) were only detected in the *TaL-LecRLK10-2D* and *TaL-LecRLK41-7A* promoters (Table 4). These patterns imply specialized regulatory adaptations in response to environmental stimuli. Growth- and development-related elements comprised 9.1% of the total. This category included the meristem-associated CAT-box, the zein metabolism-regulating O_2_-site, the seed-specific RY-element, and the endosperm expression-controlling GCN4_motif (Table 4), suggesting the involvement of *TaL-LecRLK* genes in developmental regulation throughout various growth stages. Additionally, several elements, such as ABRE, CGTCA-motif, TGACG-motif, and G-Box, were widely distributed across the promoters of various *TaL-LecRLK* homologs (Table S5; Figure S3). These conserved regulatory elements likely constitute central regulating hubs coordinating inter-pathway crosstalk during stress responses.

### 2.7. GO Analysis of the TaL-LecRLK Genes

**Figure 6:**
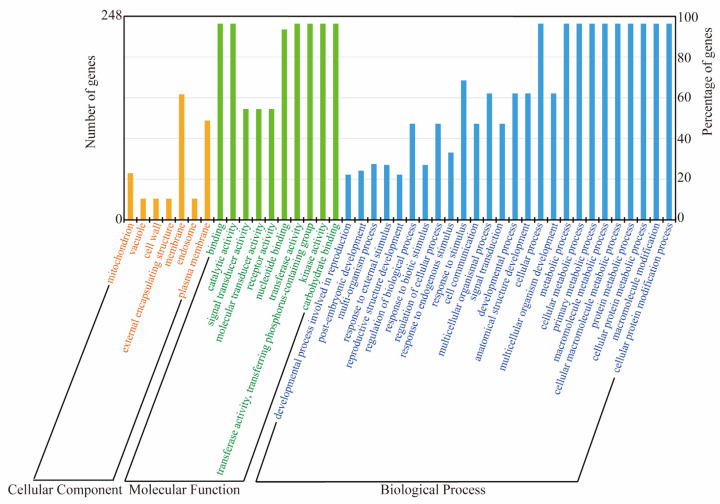
GO annotation of *TaL-LecRLK* genes, classified into biological process, molecular function, and cellular component. Supplementary Table S6 provides details.

A comprehensive Gene Ontology (GO) analysis was performed on the 248 *TaL-LecRLK* genes (Figure 6). Among them, 239 members (96.4%) were successfully annotated to 87 GO terms, of which 43 terms were statistically enriched (*p* < 0.01). These were distributed across three major categories: biological processes (26 terms), cellular components (10 terms), and molecular functions (7 terms) (Figure 6; Table S6). Within the biological process category, enriched GO terms included post-translational protein modification (GO: 0006464, GO: 0043412) and metabolic regulation (GO: 0044237, GO: 0008152), indicating potential regulatory functions in protein homeostasis and metabolic balance. Significant enrichment was also observed in cell signaling (GO: 0007165), developmental regulation (GO: 0032502), and environmental stress responses (GO: 0050896), suggesting the critical roles of *TaL-LecRLK* genes in growth and adaptation. At the molecular function level, enriched terms included kinase activity (GO: 0016301), phosphoryltransferase activity (GO: 0016772), and nucleotide binding (GO: 0000166), consistent with their roles in phosphorylation-mediated signal transduction. The enrichment of binding activity (GO: 0005488) and catalytic activity (GO: 0003824) further suggests multifunctionality in molecular interaction networks and enzymatic reactions. For cellular components, most gene products were predicted to localize to the plasma membrane (GO: 0005886) and general membrane systems (GO: 0016020). This subcellular localization aligns with their proposed functions as membrane-associated receptors in transmembrane signaling.

### 2.8. Spatiotemporal Expression Patterns of the TaL-LecRLK Genes

**Figure 7:**
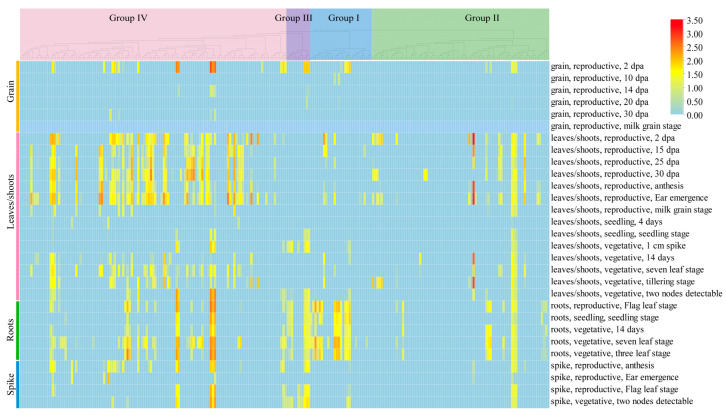
Heatmap of 248 *TaL-LecRLK* gene expression profiles at different wheat developmental stages, based on log_2_(TPM + 1) values from the wheat expression database.

To systematically analyze the spatiotemporal expression characteristics of *TaL-LecRLK* genes, RNA-seq datasets for roots, leaves/shoots, grains, and spikes of Chinese Spring wheat during both vegetative and reproductive stages were obtained from the Wheat Expression Browser (https://www.wheat-expression.com/, accessed on 10 March 2025; Table S7). A tissue-specific expression heatmap of the *TaL-LecRLK* gene family was subsequently generated based on log_2_-transformed normalized expression values (TPM+1) (Figure 7; Table S8). The expression profiling revealed that, although *TaL-LecRLK* genes were broadly expressed across various tissues, including roots, leaves/shoots, grains, and spikes, their expression patterns displayed significant developmental stage specificity. During grain development (10–30 days after flowering), the majority of genes (>99%) exhibited negligible expression (TPM < 1), although limited expression was still detectable in early grain samples collected at 2 days post-anthesis (DPA). Moreover, several genes, such as *TaL-LecRLK73-6B*, *TaL-LecRLK30-2D*, and *TaL-LecRLK11-2B*, were either lowly expressed or unexpressed in vegetative-stage leaves/shoots but became upregulated during the reproductive stage in the same tissues (Figure 7; Table S8). Notably, more than half of the *TaL-LecRLK* family members (55.6%, 138/248) showed no expression or consistently low expression levels across all sampled stages (maximum log_2_(TPM+1) < 1), implying possible functional redundancy or subfunctionalization (Table S8). Conversely, three homologous gene sets, *TaL-LecRLK20* (2A/2B/2D), *TaLecRLK38* (3A/3B/3D), and *TaL-LecRLK68* (6A/6B/6D), maintained relatively high expression levels in all sampled tissues and stages, suggesting that these genes may serve as core regulators in wheat growth and development (Table S7).

### 2.9. Abiotic Stress-Responsive Profiling of the TaL-LecRLK Genes

**Figure 8:**
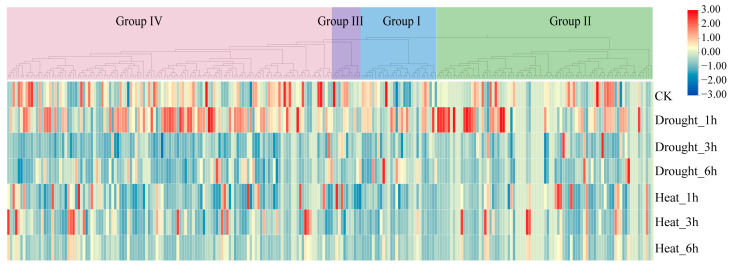
Heatmap of 248 *TaL-LecRLK* gene expression profiles in seedlings under drought and heat treatments. Expression values are the mean FPKM from three biological replicates.

To investigate the involvement of *TaL-LecRLK* genes in abiotic stress responses, one-week-old seedlings were exposed to rapid drought (filter paper dehydration) and high-temperature (42 °C) conditions. Leaf samples were collected at 0 h (CK) and 1, 3, and 6 h after stress exposure for transcriptome sequencing (three biological replicates per condition). The transcriptomic profiles of all 248 *TaL-LecRLK* genes under drought and heat stress are presented in Table S9. Expression heatmaps were generated based on the average FPKM values across replicates (Figure 8). Differential expression analysis (|log_2_FoldChange| ≥ 1, FDR < 0.01) identified 44 and 35 *TaL-LecRLK* genes that were significantly upregulated or downregulated under drought and heat stress, respectively (Figure 8; Table S10). A Venn analysis of differentially expressed genes (DEGs) across various stress durations revealed that 4 genes were persistently regulated under drought group conditions at all three time points, while 11 genes exhibited sustained responsiveness to heat stress (Figure S4). Under drought stress, the number of DEGs increased over time. Initially, gene expression was characterized by unidirectional upregulation (1 h), which transitioned into a bidirectional pattern involving both up- and downregulation by 3 h and 6 h (Table S11). These findings suggest that *TaL-LecRLK* genes may function within temporally dynamic regulatory networks during drought stress responses. A comparative analysis further revealed coordinated expression patterns among certain genes under both stress conditions. For instance, *TaL-LecRLK34-7B* and *TaL-LecRLK78-7B* showed consistent expression trends (either upregulated or downregulated) under both drought and heat treatments. In contrast, *TaL-LecRLK67-6A*, *TaL-LecRLK68-6A*, and *TaL-LecRLK16-2D* displayed opposing regulatory patterns, being upregulated under drought stress but downregulated under heat stress. These contrasting responses reflect a potential ‘synergistic and antagonistic’ dual regulatory mechanism that may enable stress-specific functional modulation through layered signal transduction pathways.

**Figure 9:**
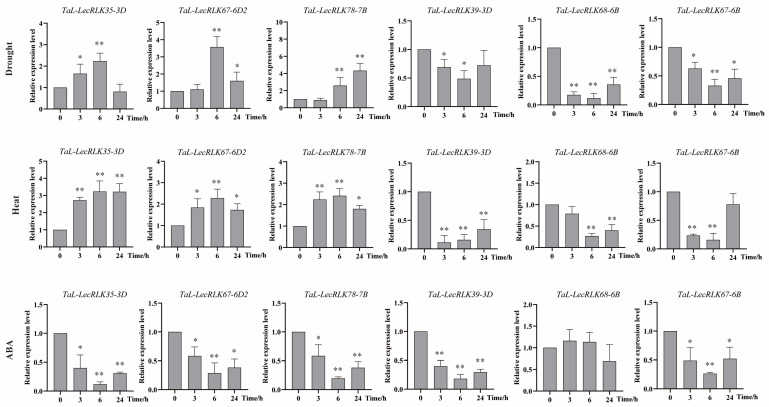
A qRT-PCR analysis of six *TaL-LecRLK* genes under drought, heat, and ABA treatments. *β-ACTIN* was used as an internal control. Mean and standard deviation (SD) values were calculated from three biological replicates. The y-axis shows relative expression; the x-axis indicates time points (0, 3, 6, and 24 h). Significant differences are indicated as follows: * *p* < 0.05; ** *p* < 0.01.

To validate the RNA-seq results, the expression patterns of six stress-responsive *TaL-LecRLK* genes were examined by qRT-PCR. Under drought stress, the upregulated genes *TaLecRLK35-3D*, *TaLecRLK67-6D*, and *TaLecRLK78-7B* reached peak expression at 6 h, 6 h, and 24 h, respectively. Meanwhile, the downregulated genes *TaLecRLK39-3D*, *TaLecRLK68-6B*, and *TaLecRLK67-6B* showed maximal suppression at 6 h (Figure 9). Under heat stress, all upregulated genes reached peak expression at 6 h, while downregulated genes displayed minimal expression at 3 h, 6 h, and 6 h, respectively. These qRT-PCR results demonstrated high consistency with the transcriptome data. Additionally, a qRT-PCR analysis under exogenous ABA treatment revealed that five of the six genes (excluding *TaLecRLK68-6B*) were significantly downregulated in a time-dependent manner (Figure 9). This finding suggests that the stress-responsive regulation of these *TaL-LecRLK* genes may proceed via ABA-independent pathways.

## 3. Discussion

L-type lectin receptor-like kinase (*L-LecRLK*) genes are known to play pivotal roles in regulating plant growth and development, responding to biotic and abiotic stresses and mediating transmembrane signal transduction processes [7]. Due to these diverse functions, *L-LecRLK* genes are promising targets for crop molecular breeding and improvement [28].

In the present study, a total of 248 wheat *L-LecRLK* genes were identified (Table S1). The prediction and analysis of sequence length, physicochemical properties, and subcellular localization revealed that the proteins encoded by the *TaL-LecRLK* gene family displayed significant heterogeneity in both structural characteristics and subcellular distribution. This diversity in sequence and location provides a molecular foundation for their broad and specialized biological functions in wheat. Additionally, we found that the number of *TaL-LecRLK* genes in wheat (248) was significantly greater than those reported in *Arabidopsis* (43) and rice (76) (Table S3), representing 5.8-fold and 3.3-fold expansions, respectively. This makes wheat the flowering plant with the largest known *L-LecRLK* gene family to date [35,36]. A further analysis revealed that *TaL-LecRLKs* are relatively evenly distributed across the A, B, and D subgenomes, with 87, 77, and 75 members, respectively (Figure 3), suggesting balanced evolutionary pressures and potential functional redundancy across subgenomes [34]. Notably, compared with the whole-genome average [5], *TaL-LecRLK* homoeologs showed a significantly higher rate of lineage-specific duplication (n:1:1/1:n:1/1:1:n; 21.8% vs. 5.7%) and a markedly lower proportion of orphan or singleton genes (1.6% vs. 37.1%) (Table 3). These findings suggest that, throughout wheat evolution, paralogous genes were retained through subfunctionalization or neofunctionalization rather than eliminated as redundant copies [37,38].

To investigate the evolutionary relationships within the *L-LecRLK* gene family, a phylogenetic analysis was conducted, classifying the *TaL-LecRLKs* into four major groups (Figure 1 and Figure S1). The classification was highly consistent with groupings observed in model species such as Arabidopsis and rice, indicating a conserved evolutionary framework across species [13,39]. Among these groups, Groups I, II, and IV exhibited notable expansions, with several subclades demonstrating monocot-specific distributions. A synteny analysis further revealed strong collinearity between monocot species (rice-wheat) (Figure S2), implying that lineage-specific functional divergence likely occurred following the monocot–dicot split. Notably, the number of Group II genes in wheat was significantly higher (83 genes, 33.5% of the wheat family) than in *Arabidopsis* (1 gene) or rice (7 genes). This pronounced expansion, likely driven by wheat’s polyploidy and intense adaptive selection under complex environmental conditions, suggests a core role for Group II L-LecRLKs in wheat’s evolution and adaptation to diverse stresses. Although Group IV contained the highest number of *L-LecRLK* genes (125 members), its relative proportion within the wheat family (50.4%) was notably lower than in Arabidopsis (83.7%) and rice (56.6%). This pattern—large absolute number but reduced relative proportion—suggests a distinct evolutionary trajectory for Group IV. Despite the lower proportional representation in wheat, the substantial number of Group IV genes implies their continued functional significance in wheat, potentially involving conserved core functions or roles adapted to monocot-specific or wheat-specific biology. Overall, these contrasting expansion patterns in Group II and Group IV likely reflect adaptations to wheat-specific selective pressures, contributing significantly to its ecological success.

Intron deletion and insertion are known important mechanisms driving gene evolution and functional diversification [40]. In this study, a high proportion of *TaL-LecRLK* genes (59.68%) were found to be intronless (Table 1; Figure 2a). Previous studies showed that intronless genes often function as rapid responders to environmental stimuli [41,42]. In Group IV, 84% of *TaL-LecRLK* genes lacked introns, suggesting a strong evolutionary conservation and possibly fundamental biological functions [43]. In contrast, Group II showed the greatest variation in intron number, indicating frequent intron gain/loss events that may have contributed to functional expansion and environmental adaptability. Conserved motif analysis also highlighted potential mechanisms of functional divergence. Core functional motifs such as Motifs 10 and 11 were widely distributed across all clades (Figure 2a,c), whereas Motifs 14 and 16 were predominantly found in Group IV, possibly due to selective loss in other clades under environmental pressures. The absence of Motif 18 in Group II, despite its high prevalence in other groups, suggested further differentiation in function. These findings align with previous studies on the wheat SnRK family [44], in which protein motif differences were closely associated with functional differentiation. Together, these results indicate that the wheat *L-LecRLK* gene family has evolved a complex genetic architecture shaped by lineage-specific expansions, intron structural variation, and selective motif retention, which may underlie its adaptation to a wide range of ecological niches.

Chromosomal distribution, gene duplication mechanisms, and evolutionary dynamics of *TaL-LecRLKs* were further analyzed. The 248 identified genes were unevenly distributed across the 21 chromosomes, with homoeologous group 2 (2A/2B/2D) exhibiting the highest enrichment (64 genes) and group 4 (4A/4B/4D) containing the fewest (16 genes) (Figure 3). These differences are hypothesized to result from genome remodeling events during hexaploid wheat evolution [45]. Gene duplication analysis indicated that whole-genome/segmental duplication (60%) and tandem duplication (19%) served as the main drivers of gene family expansion (Figure 4; Table S4). Whole-genome duplication may facilitate adaptive evolution by retaining functional gene clusters, while tandem duplication might contribute to rapid functional innovation in response to environmental challenges [46,47]. An analysis of evolutionary rates showed that all duplicated gene pairs exhibited Ka/Ks ratios < 1 (mean = 0.29), with 78% of the ratios clustered between 0.1 and 0.3 (Figure 5), indicating that purifying selection played a dominant role in preserving gene function and restricting deleterious mutations [48].

Orthology analysis, a widely used method for inferring gene function across species, was also conducted [49]. A total of 45 *L-LecRLK* orthologous gene pairs were identified between wheat and rice (Figure S2). Given that orthologous genes often retain conserved functions while acquiring species-specific traits [50], these results suggest that a subset of wheat *L-LecRLKs* may perform evolutionarily conserved roles similar to their rice counterparts.

The precise regulation of plant gene expression is mediated by specific interactions between promoter *cis*-regulatory elements and transcription factors [51]. In this study, a systematic analysis of *cis*-acting elements within the *TaL-LecRLK* gene family was conducted (Table S5; Figure S2). The results revealed the presence of three major categories of *cis*-regulatory elements in wheat *TaL-LecRLK* genes: hormone-responsive elements (49.1%), elements related to environmental adaptation (41.7%), and elements associated with growth and metabolism regulation (9.1%) (Table 4). Among these, ABA-responsive elements (ABRE) and MeJA-responsive elements (CGTCA/TGACG motifs) were particularly enriched, suggesting the potential involvement of *TaL-LecRLK* genes in response to drought and salt stress, as well as in biotic defense mechanisms through ABA and JA signaling pathways [52,53,54]. Prior studies showed that MBS elements, which serve as binding sites for MYB transcription factors, regulate drought stress responses [55]. Therefore, *TaL-LecRLK* genes containing MBS elements in their promoter regions are likely regulated by related MYB transcription factors during stress responses.

Furthermore, promoter regions of *TaL-LecRLK* genes were found to contain numerous *cis*-regulatory elements associated with growth and development (e.g., CAT-box, O2-site) and protein metabolism (e.g., GCN4_motif), implying potential roles in tissue-specific development and metabolic regulation. Spatiotemporal expression analysis indicated that *TaL-LecRLK* genes were expressed across a range of organs at different developmental stages, with particularly high expression levels during early grain development, notably at 2 DPA (Figure 7). This suggests potential involvement in young embryo formation and endosperm cell differentiation. GO enrichment analysis (Figure 6) further supported their roles in signal transduction, environmental stress responses, and the regulation of growth and metabolism. Collectively, these findings provide important clues for understanding the functional diversity and regulatory potential of the *TaL-LecRLK* gene family.

L-LecRLKs were shown to play crucial roles in mediating plant stress responses to abiotic stresses such as salinity, drought, and temperature extremes [14,15,16]. To identify stress-responsive gene candidates within the wheat L-LecRLK family, RNA-seq data were analyzed to assess expression patterns under drought and heat stress conditions. Multiple *TaL-LecRLK* genes were found to be differentially expressed in response to these stresses (Figure 8; Table S10). Under drought stress, the number of differentially expressed *TaL-LecRLK* genes increased over time, with distinct temporal expression peaks observed. Similar temporal expression trends were reported in other plant species [32,35], indicating that *TaLecRLK* genes may function in a stage-specific manner during stress response. Early-responsive genes are likely involved in rapid signal transmission, while late-responsive genes may contribute to metabolic reprogramming or the restoration of cellular homeostasis. Moreover, gene expression under drought and heat stress was not entirely consistent. For example, *TaL-LecRLK35-3D* and *TaL-LecRLK67-6B* exhibited synergistic expression patterns under drought and heat stress conditions (Table S11), suggesting that these genes may act in the same or interconnected pathways to confer stress tolerance. One possible hypothesis is that they form heterodimeric receptor complexes. In this scenario, each gene product could contribute distinct functional domains, with one subunit recognizing specific stress-associated ligands and the other initiating downstream signaling cascades. This cooperative action might enhance the sensitivity and specificity of the stress response, allowing the plant to more effectively combat multiple stressors simultaneously. Conversely, *TaL-LecRLK67-6A* demonstrated antagonistic expression under drought and heat stress conditions (Table S11), suggesting that *TaL-LecRLK67-6A* might be involved in two separate signaling branches that compete for limited cellular resources. Under heat stress, it could promote pathways that enhance thermotolerance but are detrimental to drought resistance, such as increased metabolic activity that consumes water. During drought, it may suppress these heat-related pathways to prioritize water-saving mechanisms [56]. The qRT-PCR validation confirmed the transcriptomic data and demonstrated that several differentially expressed *TaL-LecRLK* genes were generally downregulated in response to ABA treatment (Figure 9). This downregulation pattern suggests that these genes might be involved in non-canonical pathways such as JA or SA signaling and may not necessarily involve ABA-dependent mechanisms [57,58]. Previous studies demonstrated that drought stress can induce genes such as the soybean *GsSRK*, which enhances drought tolerance independently of ABA signaling [59]. Given the complexity of the regulatory networks involving the L-LecRLK family genes, future research should employ gene knockout and overexpression approaches, in conjunction with protein interaction assays, to further dissect the molecular mechanisms of *TaL-LecRLKs* and identify novel targets for improving crop stress resistance through molecular breeding.

## 4. Materials and Methods

### 4.1. Sequences’ Acquisition and Identification of the L-Type Lectin Receptor-like Kinase (L-LecRLK) Genes in Wheat

The identification of wheat *L-LecRLK* genes was performed based on the methods described previously [15,60], with some minor modifications. The complete genome and protein sequence files of wheat were downloaded from the Ensembl Plant database (https://plants.ensembl.org/index.html/, accessed on 23 November 2024) [61]. Annotated and identified L-LecRLK protein sequences from the rice genome were obtained from the Rice Genome Annotation Project website (https://rice.uga.edu/, accessed on 23 November 2024) [62].

The Hidden Markov Model (HMM) profile of the L-type LecRLK conserved domain (PF00139, Lectin_legB) and Pfam-A models were downloaded from the Pfam database (https://pfam.xfam.org/, accessed on 28 November 2024) [63]. BLASTP (E-value < 1 × e^−10^) and HMM searches were conducted using Tbtools-II software (version 2.210) [64], using rice L-LecRLK protein sequences and the PF00139 HMM profile, respectively. The results from both searches were integrated, and only the first variant was retained for downstream analysis (with three exceptions).

Domain validation was conducted using two bioinformatics tools: the batch CD-Search tool (https://www.ncbi.nlm.nih.gov/Structure/bwrpsb/bwrpsb.cgi/, accessed on 10 December 2024) [65,66] and the SMART database (https://smart.embl-heidelberg.de/, accessed on 10 December 2024) [67]. Proteins were confirmed to contain both a complete N-terminal functional domain (PF00139) and at least one conserved kinase domain (PF00069 or PF07114). Qualified genes were uniformly designated as ‘*TaL-LecRLK*’ followed by their chromosomal locations.

### 4.2. Physicochemical Properties and Subcellular Localization Prediction of TaL-LecRLKs

The basic physical and chemical properties of TaL-LecRLKs—including molecular weight, isoelectric points (*p*I), instability index, and other parameters—were analyzed using the ProtParam tool on the ExPASy Server (https://web.expasy.org/protparam/, accessed on 3 January 2025) [68]. Subcellular localization, signal peptides, and membrane classification were predicted using the Cell-PLoc 2.0 web-server (http://www.csbio.sjtu.edu.cn/bioinf/Cell-PLoc-2/, accessed on 3 January 2025) [69]. Information regarding cDNA length and amino acid number was obtained from the Ensembl Plants database [61].

### 4.3. Phylogenetic Analysis and Classification of TaL-LecRLK Proteins

Protein sequences encoded by *L-LecRLK* genes in wheat (*Triticum aestivum* L.), rice (*Oryza sativa* L.), and *Arabidopsis thaliana* were used for phylogenetic and evolutionary analyses. Genome and proteome data for rice and *Arabidopsis* were downloaded from Phytozome V13 (https://phytozome-next.jgi.doe.gov/, accessed on 23 November 2024) [70]. All *L-LecRLK* genes were retrieved using gene IDs from the three species, and their protein sequences were extracted using Tbtools-II.

Multiple sequence alignments were conducted using MUSCLE (Multiple Sequence Comparison by Log-Expectation) [71]. Phylogenetic trees were constructed using FastTree (version 2.1.11), with the maximum likelihood method [72]. The resulting tree was visualized and annotated using the iTOL v6 webtool (https://itol.embl.de/, accessed on 18 February 2025) [73]. Finally, a phylogenetic tree using 248 TaL-LecRLK protein sequences was reconstructed and used to categorize family members into distinct clades.

### 4.4. Gene Structure and Conserved Motifs’ Analysis of TaL-LecRLKs

Gene structures and conserved motifs were analyzed using two tools: GSDS2.0 (v2.0; https://gsds.gao-lab.org/, accessed on 21 January 2025) [74] for gene structure visualization and the MEME Suite (v5.5.7; https://meme-suite.org/meme/, accessed on 29 January 2025) [75] for motif detection. The MEME search was configured to identify up to 20 motifs, allowing 0 or 1 occurrence per sequence, with motif widths set between 6 and 50 amino acids.

### 4.5. Chromosomal Distribution and Homoeolog Identification of TaL-LecRLK Genes

Chromosomal locations for *TaL-LecRLK* genes were retrieved from the Ensembl Plants database [61] and visualized using Tbtools-II software [64]. Homoeologous genes were identified through phylogenetic analysis and validated using cross-referencing from the Ensembl Plants database [76,77].

### 4.6. Duplication and Syntenic Analysis of L-Type LecRLK Genes

Tandem gene clusters of *TaL-LecRLK*s were identified based on definitions and methods from previous studies [12,39]. Segmental and tandem duplication events were categorized by examining the chromosomal positions of *TaL-LecRLK* genes. Syntenic relationships within wheat and among wheat, Arabidopsis, and rice were analyzed using TBtools-II [64]. Advanced circos visualization features within TBtools-II were used to display synteny [64]. To evaluate selective pressure, Ka/Ks ratios were calculated using aKaKs_calculator [78].

### 4.7. Cis-Regulatory Element and Gene Ontology (GO) Analysis of TaL-LecRLKs

To identify potential regulatory elements, 2000 bp genomic sequences upstream of each *TaL-LecRLK* translation start codon were extracted and analyzed as promoter regions [79]. These sequences were submitted to the PlantCARE platform (https://bioinformatics.psb.ugent.be/webtools/plantcare/html/, accessed on 22 February 2025) for *cis*-regulatory element prediction [80]. The predicted *cis*-elements were systematically categorized, quantified, and visualized using TBtools-II [64].

GO annotations for *TaL-LecRLK* genes were retrieved from the agriGO v2 database (http://systemsbiology.cau.edu.cn/agriGOv2/, accessed on 6 March 2025) [81,82] and visualized using GraphPad Prism software (version 9.5).

### 4.8. Tissue Expression Profiling of TaL-LecRLK Genes

To investigate the expression patterns of *TaL-LecRLK* genes across different wheat organs, RNA-seq data representing various developmental stages were retrieved from the Wheat Expression Browser (https://www.wheat-expression.com/, accessed on 10 March 2025) [6]. Transcripts per kilobase million (TPM) values from multiple tissues and time points were extracted as measures of gene expression for *TaL-LecRLK* genes or their homologs. Expression profiles were visualized in a heatmap based on log_2_ (TPM+1) values [77], generated using TBtools-II [64].

### 4.9. Plant Cultivation, Growth Conditions, and Stress Treatments

Seeds of the wheat cultivar ‘Chinese Spring’ (*Triticum aestivum* L.) were germinated in plastic pots filled with a peat-based growth medium. Plants were maintained in growth chambers under controlled conditions: 22 °C/20 °C day/night temperatures, 60% relative humidity, and a 16-h light/8-h dark photoperiod. One week after sowing, seedlings were subjected to the following treatments: (1) Drought stress: seedlings were transferred to culture dishes lined with dry filter paper to rapidly induce water deficiency. (2) Heat stress: seedlings were placed in an illuminated growth chamber set to 42 °C. (3) Exogenous ABA application: a 100 μM abscisic acid (ABA) solution was uniformly sprayed onto the seedling leaves. Leaf tissues were harvested at 0, 3, 6, and 24 h after treatment, with three biological replicates per time point. All samples were immediately flash-frozen in liquid nitrogen and stored at −80 °C for further analyses.

### 4.10. RNA Isolation, RNA-Seq Library Preparation, and Illumina HiSeq 2000 Sequencing

Total RNA was extracted using the RNAiso Plus reagent (TaKaRa, Otsu, Japan), and first-strand cDNA synthesis was performed using an EX RT kit with gDNA remover (Zoman Biotech, Beijing, China). Three biological replicates were prepared for RNA-seq libraries. RNA quality and concentration were assessed using a NanoDrop 2000 spectrophotometer (Thermo Fisher Scientific, Wilmington, DE, USA), and RNA integrity was verified with an Agilent 2100 Bioanalyzer (Agilent Technologies, Palo Alto, CA, USA) [83].

Qualified RNA samples were submitted to Beijing Biomarker Technologies (BMKGENE, Beijing, China) for sequencing on the Illumina HiSeq 2000 platform (Illumina, San Diego, CA, USA) in PE150 mode. Raw reads were subjected to quality filtering to remove adapter sequences and low-quality reads [84]. Clean reads were aligned to the wheat reference genome (*Triticum_aestivum*.v2.1.genome.fa), and downstream analyses, including expression quantification, differential expression analysis, and functional annotation, were performed using BMKCloud tools (https://www.biocloud.net/, accessed on 21 March 2025). DEGs were identified based on fold change ≥ 2 and FDR < 0.01.

### 4.11. Quantitative Real-Time PCR (qRT-PCR) Analysis and Statistical Methods

A qRT-PCR was performed on an ABI QuantStudio 7 Flex real-time PCR system (Life Technologies, Carlsbad, CA, USA) using 2× HQ SYBR qPCR Mix (Zoman Biotech, Beijing, China) according to the manufacturer’s guidelines. Wheat *β*-actin was used as the internal control. All qRT-PCR reactions included three biological replicates. Relative gene expression level was calculated using the 2^−ΔΔCt^ method [85], and results were visualized using GraphPad Prism (v9.5). Data are presented as mean ± standard deviation (SD). Statistical analysis was conducted using one-way ANOVA, with significance levels set at * (*p* < 0.05) and ** (*p* < 0.01). Primer sequences are listed in Table S12.

## 5. Conclusions

In this study, 248 *TaL-LecRLK* genes were identified in the wheat genome, and a comprehensive analysis was conducted encompassing their phylogenetic relationships, chromosomal localization, gene structure, conserved motifs, *cis*-acting elements, and expression patterns. The *TaL-LecRLK* genes were primarily classified into four subfamilies. Segmental and tandem duplication events jointly contributed to the expansion of this gene family, with purifying selection playing a critical role in its evolutionary formation. The exon-intron structures and conserved motifs of the encoded proteins exhibited considerable diversity. *Cis*-element analysis indicated potential involvement of *TaL-LecRLKs* genes in hormonal regulation and responses to abiotic stress. Tissue-specific transcriptome data demonstrated distinct, stage-specific expression patterns. RNA-Seq analyses under drought and heat stress conditions showed that differentially expressed *TaL-LecRLK* genes displayed coordinated or antagonistic regulatory responses to different stress treatments. Furthermore, qRT-PCR validation suggested that six *TaL-LecRLKs* genes may operate through ABA-independent regulatory mechanisms. These findings lay the groundwork for future functional studies on *L-LecRLK* genes in wheat and offer new research perspectives into their evolutionary dynamics and potential biological roles.

## Figures and Tables

**Table 1:** The distribution of intron numbers across *TaL-LecRLK* gene subfamilies.

Subfamily	0 Intron	1 Intron	2 Introns	3 Introns	4 Introns	5 Introns	6 Introns	Total
Group I	10	18	1	0	0	0	0	29
Group II	28	31	15	3	2	3	1	83
Group III	6	5	0	0	0	0	0	11
Group IV	104	16	3	1	1	0	0	125
Total	148	70	19	4	3	3	1	248

**Table 2:** List of the identified motifs in TaL-LecRLK proteins.

MOTIF	ID	WIDTH
1	YLHEEWEQVVIHRDIKASNVLLDSSMNGRLGDFGLARLYDH	41
2	KRVSHDSRQGMKEFVAEVVSIGRLRHRNLVQLL	33
3	WEVEFGPHRFSYKDLFRATKGFSEKNLLGRGGFGSVYK	38
4	HVVGTMGYJAPELVRTGKATPETDVFAFGVFLLE	34
5	YDADEAELVLKLGLLCSHPDPSARPSMRQ	29
6	GYCRRKGELLLVYEYMPNGSL	21
7	WPQRYKIIKGVASAL	15
8	INDNHVGIDVNSLVS	15
9	NGNGSNRIVAVEFDT	15
10	HYVLGWSFSSDGPAP	15
11	VLPETVYVGFSAATG	15
12	GAFQNLSLISGKAMQVWVDYD	21
13	TGEVASFSTSFVFAI	15
14	JDISKLPKLPRLGPKPRSKVLEIVLPIAT	29
15	LVDWVWELYGRGAJL	15
16	GLLELTNGTSQLKGHAFHPTP	21
17	GDGMAFFLAPS	11
18	ATQINVTLAPLGVAKPARPLLSA	23
19	VACGRRPIEQNAEDN	15
20	VMQYLDGDAPLPELP	15

**Table 3:** Homoeologous genes of *TaL-LecRLKs* identified in the wheat genome.

Homoeologous Groups (A:B:D)	All Wheat Genes ^1^	All wheat *TaL-LecRLK* Genes		
		Number of Groups	Number of Genes	% of Genes ^2^
1:1:1	35.80%	24	72	29.00%
n:1:1/1:n:1/1:1:n ^3^	5.70%	27	54	21.80%
1:1:0/1:0:1/0:1:1	13.20%	15	61	24.60%
Other ratios	8.00%	18	57	23.00%
Orphans/Singletons	37.10%	-	4	1.60%
Total	99.80%	-	248	100%

^1^ According to IWGSC (2018) [5]. ^2^ Percentage calculated with 248 *TaL-LecRLK* genes, see Supplementary Table S3 for detailed information. ^3^ n > 1.

**Table 4:** Functional categorization and statistical profiling of *cis*-regulatory elements in *TaL-LecRLK* gene promoter regions.

Category	Function	Site Name	Number	Percentage for Each Category (%)	Percentage of the Total Number (%)
Hormonal responsiveness (2812, 49.1%)	abscisic acid responsiveness	ABRE	810	28.81	13.96
	MeJA-responsiveness	CGTCA-motif	739	26.28	12.73
		TGACG-motif	735	26.14	12.66
	gibberellin-responsiveness	P-box	103	3.66	1.77
		TATC-box	44	1.56	0.76
		GARE-motif	28	1.00	0.48
	auxin-responsive element	TGA-element	166	5.90	2.86
		AuxRR-core	40	1.42	0.69
	salicylic acid responsiveness	TCA-element	146	5.19	2.52
		SARE	1	0.04	0.02
Environmental adaptation (2418, 41.7%)	light responsive element	G-Box	868	35.90	14.96
		Sp1	212	8.77	3.65
		GT1-motif	152	6.29	2.62
		ACE	48	1.99	0.83
		MRE	48	1.99	0.83
		3-AF1 binding site	13	0.54	0.22
		AAAC-motif	5	0.21	0.09
		C-box	3	0.12	0.05
		4cl-CMA2b	1	0.04	0.02
	anaerobic induction	ARE	413	17.08	7.12
	drought-inducibility	MBS	261	10.79	4.50
	low-temperature responsiveness	LTR	171	7.07	2.95
	anoxic specific inducibility	GC-motif	127	5.25	2.19
	defense and stress responsiveness	TC-rich repeats	86	3.56	1.48
	wound-responsive element	WUN-motif	8	0.33	0.14
	dehydration, low-temperature, salt stresses	DRE	2	0.08	0.03
Plant growth, development, and metabolism (534, 9.1%)	meristem expression	CAT-box	235	44.01	4.05
	zein metabolism regulation	O2-site	143	26.78	2.46
	endosperm expression	GCN4_motif	52	9.74	0.90
	seed-specific regulation	RY-element	36	6.74	0.62
	circadian control	circadian	34	6.37	0.59
	cell cycle regulation	MSA-like	27	5.06	0.47
	differentiation of the palisade mesophyll cells	HD-Zip 1	7	1.31	0.12
Total			5804		100.00

## Data Availability

All data generated or analyzed during this study are included in the article and its Supplementary Materials.

## Supplementary Materials

The following supporting information can be downloaded at https://www.mdpi.com/article/10.3390/plants14121884/s1: Figure S1. Phylogenetic analysis of the 248 L-type Lectin Receptor-like Kinases (L-LecRLKs) in wheat (*Triticum aestivem* L.). Figure S2. Syntenic relationships of wheat *L-LecRLK* genes with *Arabidopsis thaliana* and *Oryza sativa*. Genomic collinearity regions between wheat and other species are indicated by gray lines. Blue and red lines highlight syntenic L-LecRLK gene pairs between wheat-Arabidopsis and wheat-rice, respectively. Figure S3. Analysis of *TaL-LecRLK* gene promoters and their *cis*-acting elements. (a) Distribution of predicted *cis*-acting elements in the promoter regions; (b) Quantification of different *cis*-acting elements in each promoter. Figure S4. Venn diagram of differentially expressed genes (DEGs) (|log_2_FoldChange| ≥ 1, FDR < 0.01) under drought and heat stress at different time points. Detailed data are provided in Supplementary Table S11. Table S1. List of 248 *TaL-LecRLK* genes identified in the wheat (*Triticum aestivem* L.) genome. Table S2. Detailed information on homoeologous gene groups of the *TaL-LecRLK* genes in wheat. Table S3. List of L-type LecRLKs in *Arabidopsis thaliana* and *Oryza sativa* genome. Table S4. Ka/Ks ratios of duplicated *TaL-LecRLK* gene pairs. Table S5. Identification and classification of *cis*-acting elements in the promoters of 248 *TaL-LecRLK* genes. Table S6. Gene ontology analysis of *TaL-LecRLK* genes. Table S7. TPM values of the 248 *TaL-LecRLK* genes in different wheat tissues and developmental stages. Table S8. Expression profiles of 248 *TaL-LecRLK* genes across diverse tissues and developmental stages (presented as log_2_(TPM+1) values). Table S9. Transcriptome sequencing data of the 248 *TaL-LecRLK* genes in wheat before and after drought and heat stress treatments. Table S10. Expression profiles of 248 *TaL-LecRLK* genes under normal and stress conditions. Mean FPKM (Fragments Per Kilobase of transcript per Million mapped reads) values calculated from three biological replicates. Table S11. Significantly differentially expressed *TaL-LecRLK* genes in stress-treated vs control groups. Table S12. Primers used for qRT-PCR in this study.
