# Supplementary material for: Comprehensive Genome-Wide Characterization of L-Type Lectin Receptor-like Kinase (L-LecRLK) Genes in Wheat (Triticum aestivum L.) and Their Response to Abiotic Stress
Source: Plants (Basel). 2025 Jun 19;14(12):1884. doi: 10.3390/plants14121884 (PMC12196811; doi:10.3390/plants14121884)
Supplement: Supplementary file 1 [file plants-14-01884-s001.zip › Supplementary files/Legends of Supplementary Figures and Tables.docx]

**Supplementary Materials:**

**Table S1.** List of 248 *TaL-LecRLK* genes identified in the wheat genome.

**Table S2.** Detailed information on homoeologous gene groups of the *TaL-LecRLK* genes in wheat.

**Table S3.** List of L-type *LecRLKs* in *Arabidopsis* and the rice genome.

**Figure S1.** Phylogenetic analysis of the 248 L-LecRLK proteins in wheat.

**Table S4.** *Ka*/*Ks* ratios of duplicated *TaL-LecRLK* gene pairs.

**Figure S2.** Syntenic relationships of wheat *L-LecRLK* genes with *Arabidopsis thaliana* and *Oryza sativa*. Genomic collinearity regions between wheat and other species are indicated by gray lines. Blue and red lines highlight syntenic *L-LecRLK* gene pairs between wheat-*Arabidopsis* and wheat-rice, respectively.

**Table S5.** Identification and classification of *cis*-acting elements in the promoters of 248 *TaL-LecRLK* genes.

**Figure S3.** Analysis of *TaL-LecRLK* gene promoters and their *cis*-acting elements. (a) Distribution of predicted *cis-*acting elements in the promoter regions; (b) Quantification of different *cis*-acting elements in each promoter.

**Table S6.** Gene ontology analysis of *TaL-LecRLK* genes.

**Table S7**. TPM values of the 248 *TaL-LecRLK* genes in different wheat tissues and developmental stages.

**Table S8.** Expression profiles of 248 *TaL-LecRLK* genes across diverse tissues and developmental stages (presented as log_2_(TPM+1) values).

**Table S9.** Transcriptome sequencing data of the 248 *TaL-LecRLK* genes in wheat before and after drought and heat stress treatments.

**Table S10.** Expression profiles of 248 *TaL-LecRLK* genes under normal and stress conditions. Mean FPKM (Fragments Per Kilobase of transcript per Million mapped reads) values calculated from three biological replicates.

**Figure S4.** Venn diagram of differentially expressed genes (DEGs) (|log_2_FoldChange| ≥ 1, FDR < 0.01) under drought and heat stress at different time points. Detailed data are provided in Supplementary Table S11.

**Table S11.** Significantly differentially expressed *TaL-LecRLK* genes in stress-treated vs control groups.

**Table S12.** Primers used for qRT-PCR in this study.
