# Supplementary figures and images for: Comprehensive Genome-Wide Characterization of L-Type Lectin Receptor-like Kinase (L-LecRLK) Genes in Wheat (Triticum aestivum L.) and Their Response to Abiotic Stress
Source: Plants (Basel). 2025 Jun 19;14(12):1884. doi: 10.3390/plants14121884 (PMC12196811; doi:10.3390/plants14121884)

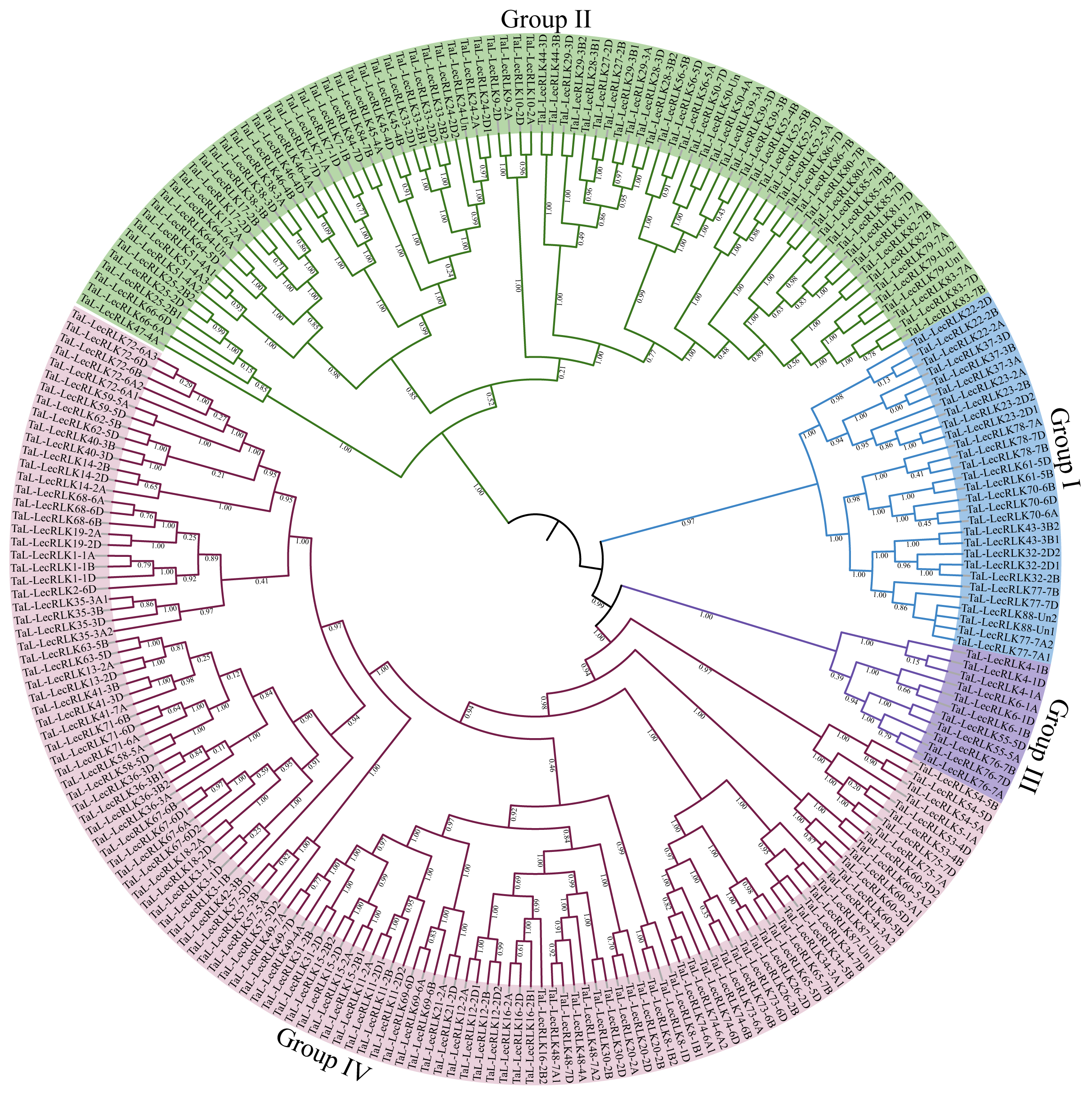

Supplement: Supplementary file 1 [file plants-14-01884-s001.zip › Supplementary files/Figure S1.tif]

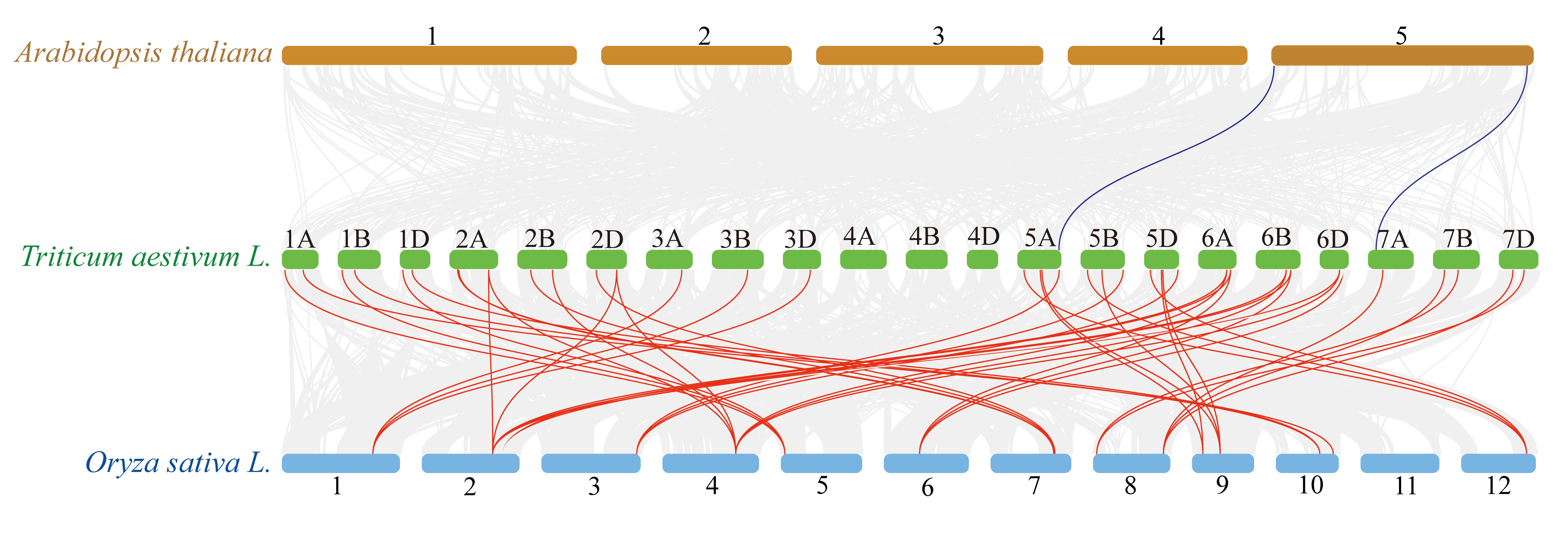

Supplement: Supplementary file 1 [file plants-14-01884-s001.zip › Supplementary files/Figure S2.tif]

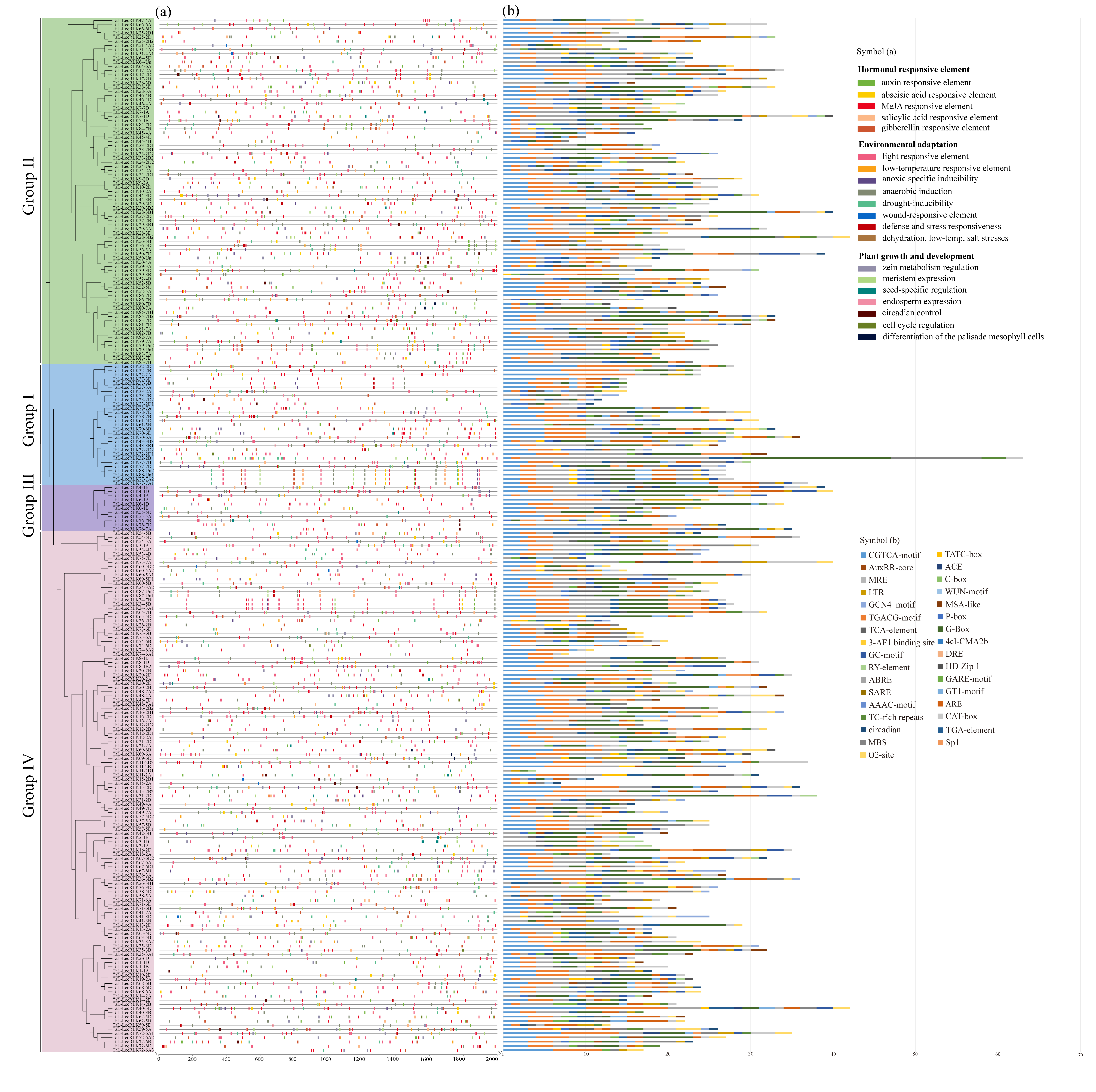

Supplement: Supplementary file 1 [file plants-14-01884-s001.zip › Supplementary files/Figure S3.tif]

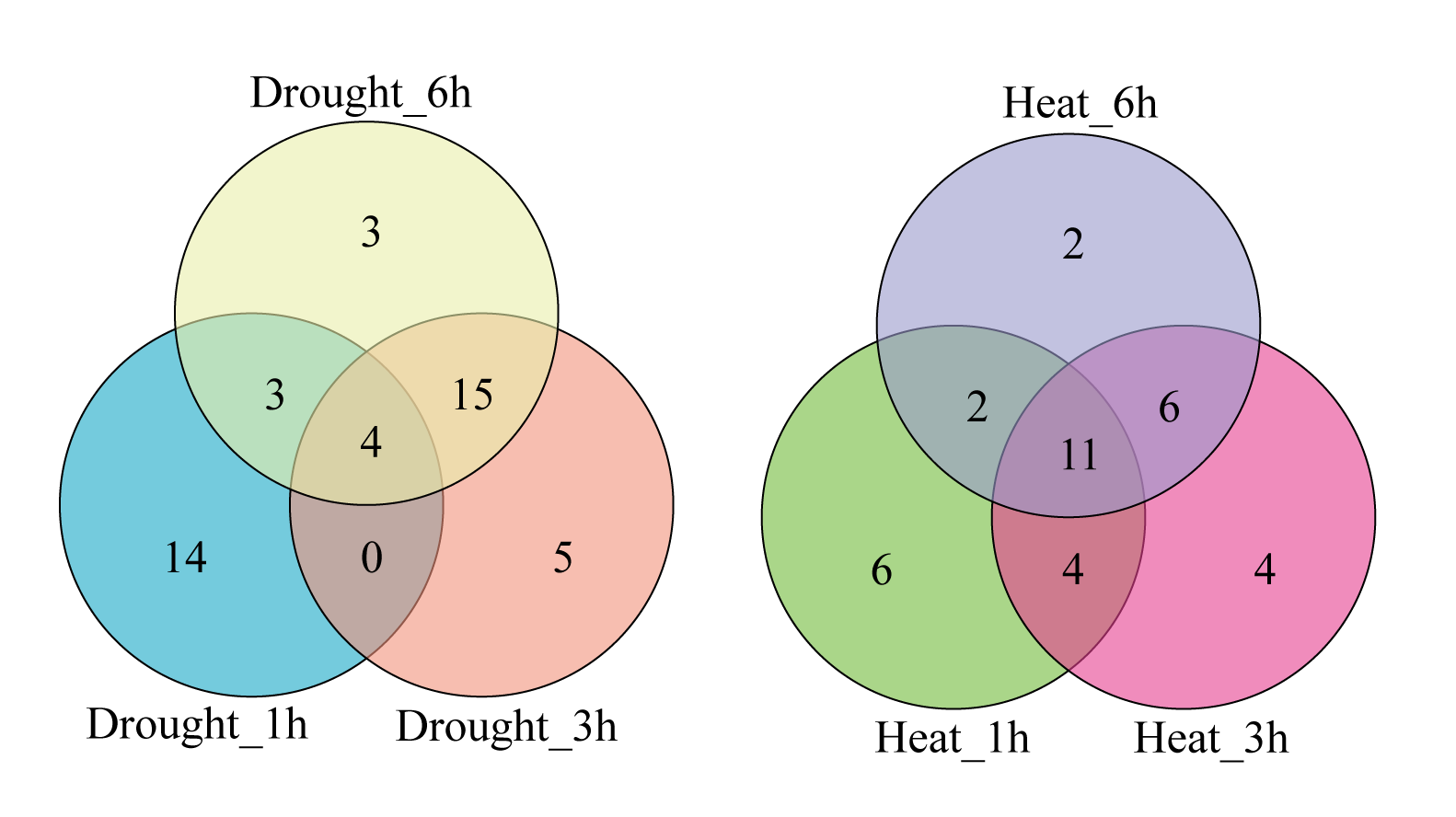

Supplement: Supplementary file 1 [file plants-14-01884-s001.zip › Supplementary files/Figure S4.tif]
